# Polymorphisms in Dopaminergic Genes in Schizophrenia and Their Implications in Motor Deficits and Antipsychotic Treatment

**DOI:** 10.3389/fnins.2019.00355

**Published:** 2019-04-17

**Authors:** Jiaen Ye, Feng Ji, Deguo Jiang, Xiaodong Lin, Guangdong Chen, Wei Zhang, Peiwei Shan, Li Zhang, Chuanjun Zhuo

**Affiliations:** ^1^Department of Psychiatry, Wenzhou Seventh People's Hospital, Wenzhou, China; ^2^Department of Psychiatry, College of Mental Health, Jining Medical University, Jining, China; ^3^Department of Psychiatric-Neuroimaging-Genetics and Morbidity Laboratory (PNGC-Lab), Nankai University Affiliated Tianjin Anding Hospital, Tianjin Mental Health Center, Mental Health Teaching Hospital, Tianjin Medical University, Tianjin, China; ^4^GHM Institute of CNS Regeneration, Jinan University, Guangzhou, China

**Keywords:** schizophrenia, dopaminergic transmission, gene polymorphism, pharmacogenetics, antipsychotic drugs

## Abstract

Dopaminergic system dysfunction is involved in schizophrenia (SCZ) pathogenesis and can mediate SCZ-related motor disorders. Recent studies have gradually revealed that SCZ susceptibility and the associated motor symptoms can be mediated by genetic factors, including dopaminergic genes. More importantly, polymorphisms in these genes are associated with both antipsychotic drug sensitivity and adverse effects. The study of genetic polymorphisms in the dopaminergic system may help to optimize individualized drug strategies for SCZ patients. This review summarizes the current progress about the involvement of the dopamine system in SCZ-associated motor disorders and the motor-related adverse effects after antipsychotic treatment, with a special focus on polymorphisms in dopaminergic genes. We hypothesize that the genetic profile of the dopaminergic system mediates both SCZ-associated motor deficits associated and antipsychotic drug-related adverse effects. The study of dopaminergic gene polymorphisms may help to predict drug efficacy and decrease adverse effects, thereby optimizing treatment strategies.

## Introduction

Schizophrenia (SCZ), characterized as a combination of hallucinations, delusions, disorganization, and other cognitive or emotional deficits, shows an average prevalence of ~1% (Kahn et al., 2015). Patients with SCZ typically present clinical symptoms in late adolescence (Gogtay et al., 2011), but the disease pathogenesis can be attributed to neurodevelopmental differences occurring at an earlier age (Kahn and Sommer, 2015). These neurodevelopmental disorders and SCZ susceptibility may be attributed to several heterogeneous genetic factors (Birnbaum and Weinberger, 2017). Recently, genome-wide association studies (GWAS) enabled the large-scale identification of novel disease-risk genes. Currently, only the Schizophrenia Working Group of the Psychiatric Genomics Consortium performed and published a GWAS (2014), reporting 108 SCZ-associated loci, which cover both glutamatergic and dopaminergic systems. This strengthened the established view of a neurotransmitter-based explanation for SCZ onset.

Neurotransmitter involvement in SCZ was substantiated by both postmortem pathological examination and *in vivo* molecular imaging. Notably, abnormal regulation of the dopamine system in SCZ was repeatedly reported, supporting the dopamine hypothesis of SCZ pathogenesis (Howes and Kapur, 2009). Dopamine system hyperactivation in SCZ patients (Grace, 2016) and the degree of dopamine release is correlated with symptom severity (Abi-Dargham, 2014). Moreover, various studies have revealed the functional roles of the dopamine system in SCZ. Dopamine transporter knockout (DAT KO) rodents showed SCZ-like behavior (Ralph et al., 2001). Furthermore, dopamine D2 receptor (D2R) upregulation in the striatum is a prominent defect in SCZ (Li et al., 2011; Simpson and Kellendonk, 2017), and dopaminergic striatal-cortical connectivity is disrupted in unmedicated SCZ patients (Horga et al., 2016). Notably, most antipsychotics mainly function via blocking D2R (van Rossum, 1966), and D2R genetic polymorphisms confer SCZ susceptibility (2014). Though studies on human genetics are frequently compromised by the population selection bias, further investigation of the dopaminergic system would benefit elucidation of pathophysiological mechanisms and development pharmaceutical interventions for SCZ.

## Dopamine Hypothesis in SCZ and Motor Disorders

Dopamine is the central modulatory system for affective and cognitive function (Grace, 2016). Aberrant dopaminergic function occurred even before puberty in mouse SCZ models (Chen et al., 2014), indicating that the dopaminergic system is involved in early disease onset. Dysfunction of dopaminergic transmission explains several SCZ symptoms. In D2R-overexpressing SCZ mice, reduced low-frequency synchrony between dopamine neurons in the ventral tegmental area and prefrontal cortex was associated with working memory deficits (Duvarci et al., 2018), and D2R overexpression in the striatum led to cognitive deficits (Kellendonk et al., 2006). Alterations in the extra-striatal dopamine receptors affected sensory input from the thalamus to the cortex (Takahashi et al., 2006). In addition to cognitive disorders, SCZ patients exhibit various motor dysfunctions, including sensorimotor deficits, dyskinesia, bradykinesia, catatonia, and psychomotor retardation (Walther and Strik, 2012). Most of those SCZ-related motor symptoms were accompanied by structural changes in premotor and motor regions, including the cerebellum, thalamus (Walther, 2015), and motor cortex (Du et al., 2019). The dopamine system is known to modulate motor behavior (Grace, 2016), and its involvement in SCZ-associated motor deficits can thus be expected. DAT KO mice show hyperlocomotion (Giros et al., 1996) and engage in stereotypic activities (Pogorelov et al., 2005). Structural evidence for locomotor dysfunction stems from altered dopaminergic circuits of basal ganglia in SCZ pathology (Perez-Costas et al., 2010). Moreover, dopamine biosynthesis disruption causes hyperactivity (Ramshaw et al., 2013). Collectively, these findings implicate the dopaminergic system in SCZ-associated motor deficits.

Behavioral studies suggest that although D2R is more likely to be involved in cognitive dysfunctions of SCZ (Papaleo et al., 2012; Jia et al., 2013), the dopamine D1 receptor (D1R) is more closely related to motor disorders (Ralph et al., 2001). However, the dopaminergic modulation of voluntary movement cannot be fully explained by this oversimplified model. Basal ganglia output is tightly controlled by direct and indirect pathways via cortical excitation on striatal neurons (Freeze et al., 2013). Briefly, dopamine synthesized in the substantia nigra is released in the striatum to activate a D1R-modulated direct pathway (Abboud et al., 2017). An indirect pathway also exists involving GABAergic neurons in the striatum and can be suppressed via D2R (Sano et al., 2013). Dopamine is essential for the homeostatic balance between these two pathways to mediate striatal input to basal ganglion nuclei and ensure normal motor function. Furthermore, activation of the D1R-mediated direct pathway initiates locomotor behavior, whereas D2R-mediated suppression of the indirect pathway can help movement maintenance (Freeze et al., 2013). SCZ patients frequently present suppressed connectivity in the substantia nigra-striatal pathway (Yoon et al., 2013), indicating aberrant motor regulation. Owing to elevated dopamine release in the striatum in SCZ (Thompson et al., 2013), motor deficits can be explained by altered dopaminergic transmission in the basal ganglia-striatum loop. Moreover, differential regulation of dendritic spine plasticity by D1Rs and D2Rs in mice was recently reported (Guo et al., 2015). These findings necessitate further comprehensive studies on the genetics and molecular mechanisms underlying the involvement of dopamine system in SCZ-related motor disorders.

## Antipsychotic Drug-Related Motor Disorders

Antipsychotics cause adverse effects, including motor disorders such as dystonia, akathisia, parkinsonism, bradykinesia, tremors, and tardive dyskinesia; these are collectively termed extrapyramidal side effects (EPS) (Leucht et al., 2009). EPS incidence varies from 7 to 32%, depending on the drug type, dosage, and demographic characteristics of patient cohorts (Novick et al., 2010). Different hypotheses have been offered for EPS, and the dopamine system has been repeatedly included in these discussions. Most of the currently used antipsychotics block dopamine receptors (Williams, 2003) based on the hyperactivity of the dopaminergic mesolimbic pathway in SCZ patients (Davis et al., 1991). Antipsychotics alter the dopamine system anatomically and functionally. Long-term antipsychotic treatment accelerated the loss of dopaminergic terminals in human basal ganglia, which has been associated with tardive dyskinesia (Seeman and Tinazzi, 2013). Therefore, the dopamine system may explain both drug sensitivity and adverse effects. Although atypical antipsychotics decrease affinity toward D2R and enhance serotonin receptor 2A binding affinity to reduce EPS incidence (Kapur and Seeman, 2001; Leucht et al., 2009), a meta-analysis revealed variable odds ratios for motor dysfunctions associated with these drugs (Leucht et al., 2013). Moreover, individual clinical trials for the same antipsychotic medication, such as clozapine, reported inconsistent EPS risk ratios (Rummel-Kluge et al., 2012). These observations further raise the possibility of individualized factors such as genetic polymorphisms that influence the adverse effects of drugs.

D2R dysfunction was reported in a rodent SCZ model (Perez and Lodge, 2012) and has become the primary target for atypical antipsychotics. The dissociation rate from the D2R determined the efficacy and adversity of antipsychotics, and accelerated dissociation permitted enhanced antipsychotic effects with less adverse effects (Kapur and Seeman, 2001). This hypothesis was supported by single-photon emission computed tomography-based human studies showing EPS correlation with D2R binding potential in the substantia nigra (Tuppurainen et al., 2010). Similarly, >80% occupancy for D2R was reported to increase EPS risk remarkably (Remington and Kapur, 1999), thus explaining the clinical correlation between lower EPS risk of clozapine and quetiapine and lower D2R occupancy (Farde et al., 1992; Kapur et al., 2000). Furthermore, another study proposed that antipsychotic effects were dependent on D2R-mediated glycogen synthase kinase−3 signaling and that EPS could be attributed to the alternative G-protein–dependent protein kinase A pathway (Su et al., 2014). Thus, both receptor binding kinetics and selective downstream signaling activation may be considered for drug development. However, an imaging study showed significant variations in D2R occupancy among patients exposed to identical dosages of antipsychotics (Miyamoto et al., 2012). This may be attributed to the effect of D2R genetic polymorphisms on binding kinetics and downstream pathway activation after antipsychotic administration. Therefore, genotype-based customized drug treatments may help to minimize side effects, including EPS, while preserving antipsychotic efficiency.

Recently, novel antipsychotics functioning as D2R agonists or antagonists, depending on dopamine levels, have been developed (Lieberman, 2004), and are expected to reduce the associated side effects. However, a recent meta-analysis showed that the EPS incidence of aripiprazole was ~17.1%, with no significant difference when compared to other atypical drugs such as clozapine, quetiapine, and olanzapine (Khanna et al., 2014; Bernagie et al., 2016). These data indicate that genetic polymorphisms, especially single nucleotide polymorphisms (SNPs) may influence the action of antipsychotics. Although a relation between genetic polymorphisms of the dopamine system and the EPS associated with D2R agonists has not yet been revealed, such an interplay may affect the binding affinity and/or downstream signaling pathway of antipsychotics, thus contributing to the observed adverse effects.

Although D1R has been strongly associated with effects of antipsychotics (Farde et al., 1992), its role in motor deficits due to antipsychotic administration cannot be neglected. Although D1R involvement in EPS development remains debatable (Coffin et al., 1989; Gerlach et al., 1996), D1R occupancy is indeed associated with EPS. For example, the classical antipsychotics haloperidol and sulpiride exhibit no apparent D1R occupancy (Farde et al., 1992), whereas clozapine shows D1R-mediated antagonist effects and low D2R occupancy, resulting in low EPS potential (Gerlach and Hansen, 1992; Gerlach et al., 1996). As D1R agonist infusion aggravates hyperactivity in SCZ rat models (Bubenikova-Valesova et al., 2009), moderate D1R blockade may reduce the EPS liability of antipsychotics. Therefore, to achieve better antipsychotic efficacy with less adverse effects, development of antipsychotics with moderate D1R blockade and partial D2R agonist activity is recommended.

## Genetic Polymorphisms in the Dopaminergic System in SCZ

Genetic polymorphisms strongly correlated with SCZ susceptibility (Harrison and Weinberger, 2004; Jablensky, 2006), and 108 SCZ-associated loci were identified (2014); however, these may include false positives due to sampling bias. Particularly, polymorphisms in the dopamine system-associated genes are repeatedly discussed (Howes et al., 2017). Dopaminergic gene mutations may regulate the formation of dopaminergic circuitry and sensitivity to antipsychotics. As yet, GWAS have failed to detect significant SCZ-associated risk sites among dopamine genes (Edwards et al., 2016); however, the potential role of dopamine genes in SCZ motor syndromes or the adverse effects of antipsychotics cannot be excluded because large-scale screenings often neglect population structures when sampling. Besides genetic regulation, microRNA-based modulation of the D2R pathway has been reported in SCZ (Hauberg et al., 2016). Akt, a serine-threonine kinase downstream from dopaminergic receptor signaling, has been associated with SCZ (Emamian et al., 2004). Dopaminergic gene SNPs are strongly related to SCZ behavioral phenotypes, and specific genotypes can be associated with different aspects of clinical symptoms (Rybakowski et al., 2006), in addition to drug sensitivity or the adverse effects of antipsychotics.

In the next section, we will briefly discuss the implications of polymorphisms in three major dopaminergic genes, catechol-O-methyltransferase (*COMT*), DAT (*SLC6A3*), and D2R (*DRD2*) in SCZ pathology, with particular interest in SNPs loci.

COMT, a catabolic enzyme involved in dopamine metabolism, effectively removes dopamine from the synaptic cleft to terminate its actions (Tunbridge et al., 2004). COMT is linked with altered prefrontal functions (Meyer-Lindenberg et al., 2006) and prefrontal-midbrain connections that are closely related to SCZ (Meyer-Lindenberg et al., 2005), and affects dopaminergic flux in the prefrontal cortex (Tunbridge et al., 2006). The *COMT* gene is located on human chromosome 22q11.2, an SCZ-associated region (Owen et al., 2004), and its microdeletion increases SCZ risk (Murphy, 2002). In addition to this microdeletion, *COMT* polymorphisms also contribute to SCZ etiology. The mutation of the haploblocks of COMT gene is closely associated with COMT functions. Within this region, Valine (Val) to methionine (Met) substitution at the rs4680 loci of *COMT* has been widely studied. In particular, the homozygous Val allele showed remarkably reduced cortical dopamine activity (Schacht, 2016). An early study reported a correlation between neuromotor performance and *COMT* Val158Met SNP (Galderisi et al., 2005). Recently, increased recruitment of supplementary motor area was reported in SCZ patients with Met homozygotes, compared to those with Val homozygotes (Lopez-Garcia et al., 2016). Tardive dyskinesia was shown to be associated with *COMT* GG genotype (Srivastava et al., 2006). These results suggest that *COMT* gene SNP may help to predict motor deficits in SCZ patients and antipsychotic treatment-associated adverse motor effects.

As dopamine transporters are abundantly distributed in striatal structures, COMT may not exert primary effects on dopamine metabolism in these subcortical regions. The tandem repeat polymorphism of the DAT gene has been related to midbrain activity (Schott et al., 2006), and DAT KO mice showed decreased cortical spine density (Kasahara et al., 2015). DAT KO mice also presented hyperlocomotion (Giros et al., 1996) and stereotypic activities (Pogorelov et al., 2005), which were rescued by psychostimulant treatment (Trinh et al., 2003). Similarly, DAT knockdown mice showed higher extracellular dopamine concentration and presented hyperactivity in a novel environment (Zhuang et al., 2001). Different distribution patterns of DAT genotypes exist in SCZ patients, compared to healthy cohorts (Persico and Macciardi, 1997). A site-specific DAT gene mutation revealed altered dopamine transmission and locomotor abnormalities (Speca et al., 2006). Functional imaging showed that DAT gene SNPs are associated with the cortico-thalamus-caudate circuit functions (Meda et al., 2010), indicating the potential behavioral relevance of these genetic polymorphisms. Clinical evidence showed that DAT gene polymorphisms affected verbal and visuospatial working memory in SCZ patients (Zilles et al., 2012) and were potentially related to antipsychotic treatment resistance (Bilic et al., 2014). Furthermore, DAT genotype was found to be associated with susceptibility to haloperidol-induced EPS (Zivkovic et al., 2013); this may be attributed to the altered temporal gyrus-cingulate-premotor patterns due to DAT SNPs (Meda et al., 2010).

A direct relation between D2R gene polymorphism and EPS has not yet been revealed (Tybura et al., 2014). However, D2R mRNA downregulation was reported after clozapine or haloperidol treatment in rats (Lipska et al., 2003). Compared to individuals homozygous for the A2 allele of D2R, A1(T) allele carriers showed reduced striatal D2R-specific binding (Eisenstein et al., 2016). The D2R binding affinity of antipsychotics can affect their efficacy and EPS. Therefore, we speculated that D2R gene SNPs are involved in these processes. Further studies are warranted to investigate drug resistance or adverse effects associated with antipsychotics in conjunction with D2R genotype screening. In addition to single gene regulation, epistatic regulation may be involved in SCZ pathogenesis and the associated phenotypic variance, and the accumulation of small effects from individual genetic polymorphisms can exert prominent effects. The variable number of tandem repeats (VNTR) of the 3′ untranslated region of the DAT gene and *COMT* Val158Met SNP interacted to modulate the activity of cortical regions, including the left supramarginal gyrus and right orbital gyrus, in SCZ patients (Prata et al., 2009), providing an example of combined effects of multigene polymorphisms on dopamine function. Furthermore, polymorphisms in a network of dopaminergic genes, including DAT, D2R, and COMT, increase SCZ susceptibility (Talkowski et al., 2008). The dopamine system may also interact with the glutamate system. DAT gene polymorphism and glutamate metabolic enzymes coregulate executive functions between striatum and para-hippocampus (Pauli et al., 2013). Collectively, dopaminergic gene polymorphisms can be prominent contributors to the cognitive and motor dysfunctions in SCZ.

## Genetic Polymorphisms in the Dopamine System in SCZ and Drug Targets

Pharmaceutical research for SCZ faces major challenges and requires a comprehensive understanding of the genetics and neural circuits involved (Pratt et al., 2012). SNPs may influence the effect of antipsychotics. The *COMT* Val158Met substitution at the rs4680 loci effectively predicted dopaminergic drug effects, as the COMT inhibitor improved cognitive functions Val homozygotes, whereas the antipsychotics were more effective in Met homozygotes (Zhuo et al., 2019). Protein structure and function studies revealed that Val-Met substitution disrupts enzyme stability, leading to suppressed dopamine clearance and higher dopamine activity (Chen et al., 2004). Hence, *COMT* SNP analysis can be used to predict antipsychotic effects. Conversely, Met homozygotes showed better response and more significant behavioral improvements after treatment with the D2R partial agonist, aripiprazole (Kaneko et al., 2018). These two results indicate the complicated homeostatic balance of the dopamine system and the potential benefit of genetic screening prior to drug administration. Recently, the “pharmacogenetics” approach has been adopted to include D2R gene polymorphisms in predicting positive and adverse drug effects (Shen et al., 2009; Giegling et al., 2013; Blum et al., 2014). Patients with the C/C genotype at the C957T SNP loci of D2R presented a relatively poor response to aripiprazole (Shen et al., 2009), resulting in a better response in A1 allele carriers of the D2R gene (Miura et al., 2012). Such phenomena can be explained by the altered binding affinity for antipsychotics due to D2R gene polymorphisms.

Recently, genome-wide screening has provided insights for novel drug targets (Schubert et al., 2014), and pharmacogenetics can be used to predict the adverse effects of antipsychotics. Although one study recruiting 191 SCZ patients failed to identify an association of EPS with polymorphisms of the D2R, DAT, or COMT genes (Tybura et al., 2014), another study indicated the predictive power of the DAT rs2975226 SNP in the response to clozapine (Xu et al., 2010). This discrepancy between may be caused by the involvement of different loci, as the DAT VNTR does not influence antipsychotic drug-induced EPS (Lafuente et al., 2007). *COMT* A-G haplotype at the A278G loci causes higher EPS susceptibility than the A-A haplotype does (Lafuente et al., 2008). EPS liability after haloperidol treatment almost doubled with DAT 9/10 and COMT Val158Met SNP (Zivkovic et al., 2013). In addition to the dopaminergic pathway genes, metabolic genes, such as those involved in the mammalian target of rapamycin pathway, are also associated with EPS susceptibility and antipsychotic response (Mas et al., 2015). Thus, genetic polymorphisms of the dopamine system are potentially correlated with EPS; however, the detailed molecular mechanisms remain unclear.

Other dopaminergic genes may also contribute to drug effects. The S/S genotype and S allele of the dopamine 3 receptor (D3R) gene are potentially associated with resistance to atypical antipsychotics in SCZ patients (Szekeres et al., 2004). The dopamine 4 receptor (D4R) was found to be associated with tardive dyskinesia syndromes in SCZ subjects (Srivastava et al., 2006). In addition to the protein-coding region mutations that alter protein structure and function, mutations in noncoding regions may also affect SCZ susceptibility. Several SNP loci in Armadillo repeat gene deleted in Velo-Cardio-Facial syndrome (*ARVCF*), downstream of *COMT*, potentially regulate SCZ risk (Mas et al., 2009). Thus, the complex relation between dopaminergic genes and antipsychotic effects necessitates further large-scale genomic screenings to better understand the regulatory network for gene polymorphism in the dopaminergic system in SCZ. These studies will aid prediction or evaluation of the adverse effects of antipsychotics.

## Dopaminergic Gene SNPs and Antipsychotic Drug-Associated Motor Deficits

Although pharmacogenetics enabled investigating the relation between antipsychotic drug effects and dopaminergic gene polymorphisms, inconsistent or even contradictory results are frequently obtained. Some studies reported no association between EPS and gene polymorphisms in D2R, DAT, and COMT genes or other dopamine metabolic or transporter genes (Tybura et al., 2014) (Lafuente et al., 2007; Gassó et al., 2010; Xu et al., 2010). Specifically, D2R amino acid variants are not the major predictors for adverse effects of antipsychotics (Kaiser et al., 2002). Notably, serotonin receptor genes were associated with antipsychotic therapy-associated EPS (Gunes et al., 2007). Conversely, the correlation between dopaminergic gene SNPs and the adverse effects of antipsychotics has been reported. One D3R SNP site (rs167771) was associated with acute EPS induced by antipsychotics (Gassó et al., 2009), and these EPS are more likely contributed by SNPs within regulatory regions and introns (Gassó et al., 2011). EPS after haloperidol treatment was associated with the 9/10 genotype of the DAT gene or the Val158Met genotype of *COMT* (Zivkovic et al., 2013). This was further supported by a report on higher EPS risk in individuals with the *COMT* A-G haplotype (Lafuente et al., 2008) and those with the 9-repeat allele of DAT1 VNTR (Güzey et al., 2007). Moreover, D2R gene SNPs contributed to EPS risk (Mas et al., 2016). Two independent studies revealed that A1 allele of the D2R gene was associated with increased EPS risk (Hedenmalm et al., 2006; Güzey et al., 2007). Similarly, increased EPS susceptibility was observed in individuals with the −141C Del allele of the D2R gene (Nakazono et al., 2005). Thus, considering such contradictory evidence, the role of dopaminergic gene SNPs in antipsychotic drug-related adverse effects, including motor disorders, needs to be investigated further.

We believe that the adverse effects of antipsychotics can be further interpreted in a systemic view of dopamine homeostasis, because SCZ onset and progression affect different aspects of dopamine transmission. COMT is crucial for cortical dopamine degradation to terminate dopamine action within the synaptic cleft. COMT being a major dopamine catabolic enzyme, *COMT* SNP may cause altered dopamine clearance levels, influencing antipsychotic effects and motor disorders. DAT is responsible for dopamine transport, and DAT expression level and protein structure may alter dopaminergic transmission strength. Dopamine targets and binds specific receptors from D1R to D4R, which have region-specific patterns (Laurier et al., 1994), thus influencing various aspects from mental functions to locomotor activities via differential modulation of downstream signaling pathways. The dopamine system can also modulate the excitatory-inhibitory balance of complete neural networks, rather than simply altering the activity of a single neuron (Birnbaum and Weinberger, 2017). Therefore, a complex interaction between different neurotransmitter systems can be expected in SCZ. Future studies of polymorphisms in genes involved in the dopamine system provide insights into the molecular pathway and enable adverse effect prediction.

Studies on molecular mechanisms of the dopamine system in SCZ pathogenesis and the adverse effects of antipsychotics mainly include investigation of receptor binding affinity and the downstream signal transduction pathways involved. Recently, G-protein coupled receptor dimerization was suggested in dopaminergic regulation. D1R and D2R can form receptor heterodimers (Dziedzicka-Wasylewska et al., 2006), which can be dissociated by clozapine (Faron-Górecka et al., 2008). These D1R-D2R heterodimers exist in macaque models and were enhanced by dopamine depletion (Rico et al., 2017). Studying the effects of antipsychotics on D1R-D2R dimerization may help to elucidate the molecular mechanisms underlying antipsychotic drug action (Dziedzicka-Wasylewska et al., 2008). However, whether polymorphisms of the dopamine receptor genes can affect the association/dissociation kinetics of these receptor dimers, remains unclear.

## Conclusions

**Figure 1:**
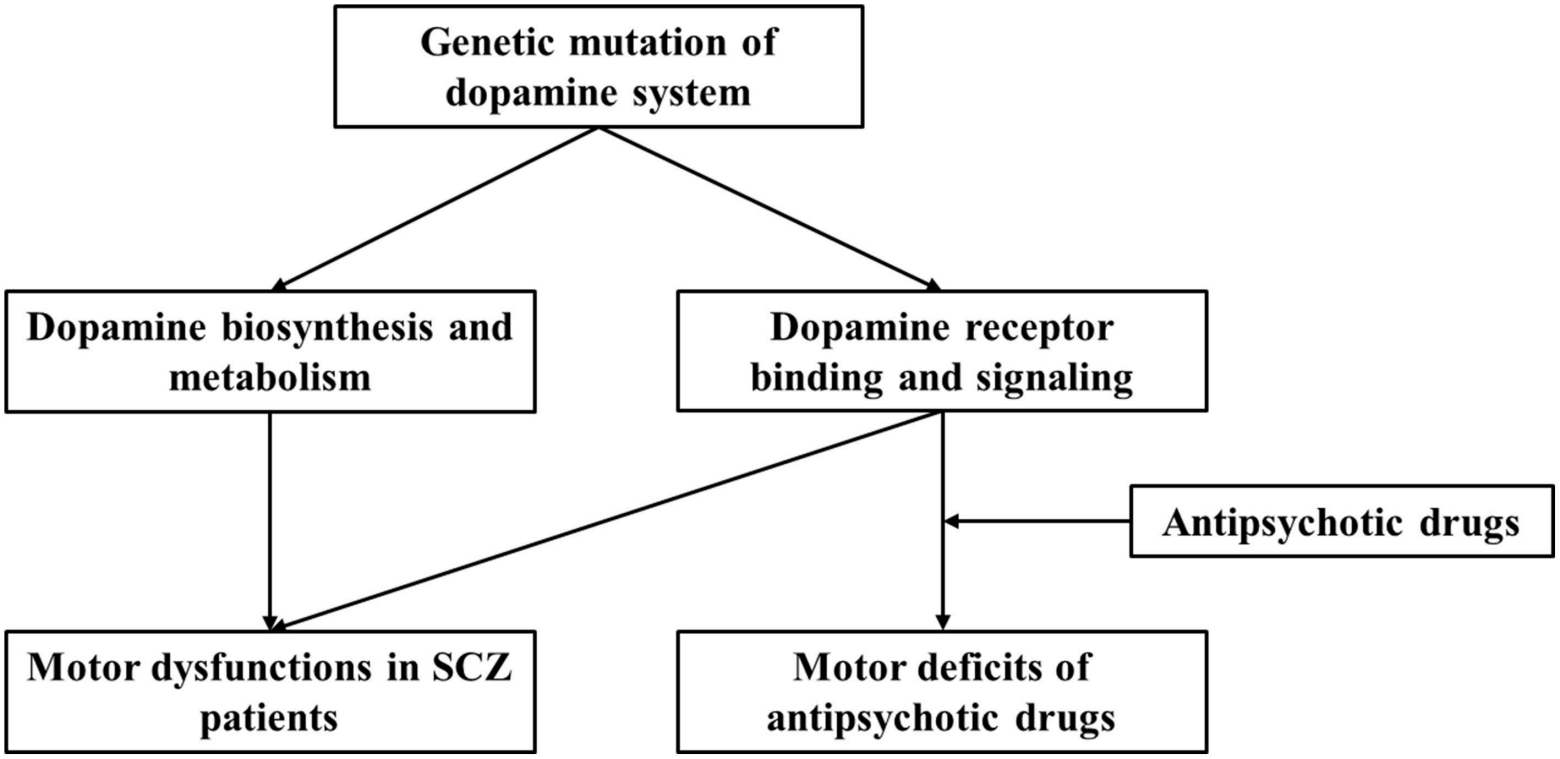
Current working model for regulation of dopaminergic system and the effect of genetic polymorphism on motor dysfunction and antipsychotic drug-related motor deficits in SCZ patients.

We present a brief review of the relation of dopaminergic genes with SCZ pathogenesis and antipsychotic treatment-related adverse effects. We focused on motor deficits associated with SCZ or antipsychotic drug administration and the role of dopamine system and gene polymorphism-based modulation. We propose that dopaminergic gene polymorphisms influence both SCZ-associated motor deficits and antipsychotic drug-induced motor dysfunction (Figure 1). More importantly, variations across individuals contribute to different SCZ susceptibilities, symptoms, responsiveness to drugs, and adverse effects, including motor disorders. However, publication bias and variation across demographic structures are the limitations involved in studies on human genetics. Moreover, both GWAS and human candidate gene studies have their limitations, as GWAS can suffer from ignorance of environmental interaction and functional relevance, and candidate gene study cannot identify synergistic effects among multiple genes. In this study, we only discuss dopamine related loci and SNPs from published data, and thus cannot cover those genetic interactions with other genes, although such relationship may exert important roles for brain functions. Taken together, it is essential to combine genetic screening data with clinical manifestations and use animal studies for mechanistic substantiations. Nevertheless, the analysis of gene polymorphism patterns can be useful in predicting disease risk and evaluating antipsychotic actions and potential adverse effects.

## Hypothesis

Dopamine system aberrations are strongly associated with schizophrenia symptoms, including motor disorders, and both antipsychotic action and adverse effects, including motor disorders. Thus, investigating dopaminergic gene polymorphisms may help to predict drug efficacy and decrease adverse effects, thereby optimizing treatment strategies.

## Author Contributions

JY, FJ, DJ, XL, GC and WZ collected research articles and prepared necessary materials for writing this review. LZ wrote this manuscript with the help from CZ and PS. CZ, LZ and PS revised the manuscript. All authors have approved this manuscript before submission.

### Conflict of Interest Statement

The authors declare that the research was conducted in the absence of any commercial or financial relationships that could be construed as a potential conflict of interest.

## Abbreviations

COMT: catechol-O-methyltransferase
D1R: dopamine D1 receptor
D2R: dopamine D2 receptor
D3R: dopamine 3 receptor
DAT: dopamine transporter
EPS: extrapyramidal side effects
GWAS: genome-wide association studies
KO: knockout
SCZ: schizophrenia
SNP: single nucleotide polymorphism
VNTR: variable number of tandem repeats
